# Spatiotemporal Study of COVID-19 in Fars Province, Iran, October-November 2020: Establishment of Early Warning System

**DOI:** 10.1155/2022/4965411

**Published:** 2022-05-30

**Authors:** Ali Semati, Azimeh Zare, Marjan Zare, Alireza Mirahmadizadeh, Mostafa Ebrahimi

**Affiliations:** ^1^Non-Communicable Diseases Research Center, Shiraz University of Medical Sciences, Shiraz, Iran; ^2^Department of Anesthesiology, Gerash University of Medical Sciences, Gerash, Fars, Iran; ^3^Maternal-Fetal Medicine Research Center, Shiraz University of Medical Sciences, Shiraz, Iran; ^4^Communicable Disease Control Center, Shiraz University of Medical Sciences, Shiraz, Iran

## Abstract

**Background:**

Using time series and spatiotemporal analyses, this study aimed to establish an Early Warning System (EWS) for COVID-19 in Fars province Iran.

**Methods:**

A EWS was conducted on (i) daily basis city-level time series data including 53 554 cases recorded during 18 February–30 September 2020, which were applied to forecast COVID-19 cases during 1 October–14 November 2020, and (ii) the spatiotemporal analysis, which was conducted on the forecasted cases to predict spatiotemporal outbreaks of COVID-19.

**Results:**

A total of 55 369 cases were forecasted during 1 October–14 November 2020, most of which (26.9%) occurred in Shiraz. In addition, 65.80% and 34.20% of the cases occurred in October and November, respectively. Four significant spatiotemporal outbreaks were predicted, with the Most Likely Cluster (MLC) occurring in ten cities during 2–22 October (*P* < 0.001 for all). Moreover, subgroup analysis demonstrated that Zarrindasht was the canon of the epidemic on 6 October (*P*=0.04). As a part of EWS, the epidemic was triggered from Jahrom, involving the MLC districts in the center, west, and south parts of the province. Then, it showed a tendency to move towards Zarrindasht in the south and progress to Lar in the southernmost part. Afterwards, it simultaneously progressed to Fasa and Sepidan in the central and northwestern parts of the province, respectively.

**Conclusion:**

EWS, which was established based on the current protocol, alarmed policymakers and health managers on the progression of the epidemic and on where and when to implement medical facilities. These findings can be used to tailor province-level policies to servile the ongoing epidemic in the area; however, governmental level effort is needed to control the epidemic at a larger scale in the future.

## 1. Introduction

The surveillance systems for infectious diseases are encouraging well-developed techniques that can warn them by detecting the exact time and place of disease occurrence and, subsequently, targeting interventions and resources to more risky areas [1]. Some infections follow space and time trends for which Early Warning Systems (EWS) can be useful tools to find the potential outbreaks [2]. EWS are alert systems to predict epidemic outbreaks in the region. In the 1910s, Captain S.R Christophers from the British army set a system to predict malaria in India. Since then, it has been used as a useful tool to aid infected nations, make health policies, and manage services to the infected area [1]. The epidemic statue of *SARS-CoV-2* disease (COVID-19) was officially declared by the World Health Organization in January 2020 [3, 4]. What makes it important to set a EWS for ongoing COVID-19 epidemic is that it could early notify the policy makers and health managers on the time and location of the outbreaks, so that they could focus on more needy areas and dedicate more medical facilities. Finally, people are helped with less COVID-19 threat, infections, and death.

The question is that, using COVID-19 time series data, if the current study could establish a EWS for future COVID-19 outbreaks, the surveillance system could act timely, prioritize times and locations for targeting interventions, allocate resources more efficiently, and improve decision-making in the area.

Time series data refer to a sequence of successive naturally temporal data, which are equally spaced in points of time. A time series analysis consists of methods for analyzing time series data and forecasting the future values based on the previously observed values [5]. Spatial and Temporal Scan (SaTScan) modeling discovers more about the space and time of an event occurrence. Space-time permutation scan statistic, which is derived from SaTScan, was first introduced by Martin Kulldorff in 2015 [6]. This model aims at determining whether the cases are distributed uniformly over time and space. The statistic utilizes scanning windows moving systematically across the study area to identify the clusters of cases. This modeling helps surveillance systems decide timely on more at risk areas [7]. Using one model output as another mode input, this study could develop a protocol of EWS in the area by (i) forecasting the COVID-19 cases, (ii) discovering the future space-time outbreaks of COVID-19, (iii) predicting the progression of epidemic, and (iv) having more control on the epidemic.

## 2. Methods

### 2.1. Study Design, Data Source, and Study Variable

Daily basis city-level time series data including 53 554 cases recorded in 26 cities of Fars province, Iran, during 18 February–30 September 2020 (225 days) were applied to forecast COVID-19 cases during 1 October–14 November 2020 (45 days). The cases were obtained from 44 COVID-19 sampling centers affiliated to Shiraz University of Medical Sciences (SUMS). All data were reported to SUMS either online, https://www.coronalab.sums.ac.ir, or by manual registration. There were no exclusion criteria, meaning that all cases were included in the study. The COVID-19 cases were the positive cases confirmed by real-time Reverse Transcription Polymerase Chain Reaction (RT-PCR). Additionally, the geographical coordinates of the locations were obtained through Google-Earth (US Dept. of State Geographer 2021) based on the latitude-longitude coordinate system.

### 2.2. Case Study and Area

Fars province, the fourth most populated (4 852 274 dwellers) and widespread (122 608 km^2^) province amongst 31 provinces in Iran, is located at 27°3′ and 31°40′ northern latitude and 50°36′ and 55°35′ eastern longitude in south of Iran. It includes 26 cities, with Shiraz, the fifth most populated city in Iran, being the capital. Marvdasht, Jahrom, Fasa, Kazerun, Darab, Firoozabad, Lar, and Abade, also, are the most populated areas (more than 100 00 dwellers per capita). 67.6% of the province are urban dwellers (urban areas and suburbs), 31% are villagers (small towns and rural areas), and the rest are nomads (no fixed habitation who regularly move to and from the same areas breeding livestock) [8].

### 2.3. Statistical Analysis

Mean difference and Std. Error of difference were used to describe quantitative variables, and frequency (relative frequency) was used to describe qualitative variables.

The distribution of COVID-19 cases across cities and times are tested using independent-sample one way ANOVA and pair wise comparisons (LSD test). Additionally, time series analysis including (i) Akaik Information Criterion (AIC)/Bayesian Information Criterion (BIC), which are the estimators of the relative quality of time series models comparative to other models for a given set of data, (ii) Auto Correlation Function (ACF)/Partial Auto Correlation Function (PACF) sample/model, which assess the conformity of the observed and fitted pattern in the data, and (iii) residual analysis, which evaluates the presumptions of white noise parameter, was used to forecast the COVID-19 cases during 1 October–14 November 2020 (45 days). A set of time series models were applied, and the best ones were selected based on lower AIC/BIC scores, ACF/PACF conformity, and residual analysis.

Descriptions on time series models:

A simple additive decomposed time series model is
  (1) $$\begin{array}{c} x_{t}=m_{t}+s_{t}+e_{t}, \end{array}$$
*m*_*t*_ is the trend, *s*_*t*_ is the seasonality, and *e*_*t*_ is the error or random white noise.
To be used to predict well founded values, a time series must be stationary, meaning that it should have a constant mean and variance across the time series. So, in order to allow forecasting, several time series models can be created including Autoregressive Model (AR), Moving Average Model (MA), and Autoregressive Moving Average (ARMA) model, which are used on stationary time series. Seasonal Autoregressive Moving Average (SARMA) model, however, is used for nonstationary time series.
Autoregressive Model: AR (*p*)
AR model uses observations from previous time steps as input to a regression equation to predict the value at the next step. It takes one argument, *p*, which determines how many previous time steps will be input. The order, *p*, can be determined by looking at the PACF. The PACF gives the partial correlation of a time series with its own lagged value, regressed of the time series at all shorter lags.
Moving Average Model: MA (*q*)
MA model is a time series model that accounts for very short-run autocorrelation. Basically, it states that the next observation is the mean of every past observation. The order of MA model, *q*, can usually be estimated by looking at ACF plot.
Autoregressive Moving Average: ARMA (*p*, *q*)
ARMA and ARIMA models are simply a combination of an AR model and MA model.
Seasonal Autoregressive Moving Average model: SARMA_*s*_(*p*, *q*)
SARMA model is an extension of ARMA model. The only difference is that a seasonal component is added now. *s* is the number of periods in the season. SARMA can adjust a nonstationary time series by removing trend and seasonality. [9].

Appling time series forecasted cases in spatiotemporal analysis, high-rate clusters were detected considering the minimum and maximum temporal sizes that were equal to one day and 50% of the study period, respectively. The minimum and maximum spatial sizes were also equal to two cases and 50% of at risk population, respectively. Temporal data were checked to ensure that all the cases were within the specified temporal study period. In addition, geographical data check was done to ensure that all the observations were within the specified geographical area. No geographical overlap and subgroup analysis were used.

R v.3.6.3, ITSM 2002, ArcGIS v. 10, and SaTScan software were applied for data analysis. Significance level was considered 0.05 for all tests.

### 2.4. Validation Study

A validation study using real data from 1 October–14 November 2020 was done to assess the accuracy of the results.

### 2.5. Ethical Statement

All ethical steps including data collection and analysis as well as reporting the results were in accordance with the standards approved by the Ethic Committee of the Ministry of Health, Treatment, and Medical Education (IR.SUMS.REC.1399.574). The work processes were anonymous, and the results were reported to the study participants.

## 3. Results

### 3.1. Distributions of 53 554 COVID-19 Cases in Fars Province by City and Month, February 18–September 30, 2020

From 53 554 COVID-19 cases, the maximum and minimum cases happened in Shiraz (53.49%) and Sarvestan (0.2%), respectively. In addition, the maximum and minimum cases happened in July (24%) and February (0.3%), respectively. The distribution of 53 554 COVID-19 cases by city and month is shown in Tables 1 and 2.

COVID-19 cases were not distributed uniformly across cities (test statistics = 170.83, d*f* = 25, *P* < 0.001) and months (test statistics = 21.3, d*f* = 7, *P* < 0.001) in Fars province during February 18–September 30, 2020. Pairwise comparisons of 53 554 COVID-19 cases by city and month have been shown in Tables 3 and 4.

COVID-19 cases were significantly higher in Shiraz than those in 25 other cities (*P* < 0.05 for all). Jahrom had more cases than Farashband, Khonj, Darab, Rostam, Kharame, Zarindasht, Sarvestan, Firoozabad, Ghirkarzin, Marvdasht, Lamerd, Mamasani, and Sepidan (*P* < 0.05 for all); however, fewer cases were seen in Jahrom compared with Lar (*P* < 0.05). In addition, Safashahr has lower cases compared with Fasa, Jahrom, Kazerun, Marvdasht, and Lar (*P* < 0.05 for all). Also, there were more cases in Fasa than those in Neireez, Abade, Arsenjan, Stahban, Eghlid, Bavanat, Pasargad, Kharame, Farashband, khonj, Darab, Rostam, Zarindasht, Sarvestan, Firoozabad, Ghirkarzin, Marvdasht, Lamerd, Sepidan, and Mamasani (*P* < 0.05 for all); there, however, were fewer cases in Fasa compared with Lar (*P* < 0.05). Moreover, fewer cases were seen in Neireez, Arsenjan, Stahban, Eghlid, Pasargad, and Bavanat than those in Jahrom, Kazerun, Marvdasht, and Lar (*P* < 0.05 for all, respectively). Furthermore, the cases were lower in Kharame, Khonj, Rostam, Ghirkarzin, Sarvestan, and Farashband than those in Kazerun, Marvdasht, and Lar (*P* < 0.05 for all, respectively). Additionally, the cases were lower in Abade than those in Jahrom, Kazerun, and Lar (*P* < 0.05 for all). Likewise, the cases were lower in Darab, Firoozabad, and Zarrindasht than those in Kazerun, and Lar (*P* < 0.05 for all, respectively). Besides, the cases were more in Kazerun than those in Lamerd, Mamasani, and Sepidan (*P* < 0.05 for all); however, less cases were seen in Kazerun compared with Lar (*P* < 0.05). Also, the cases were higher in Marvdasht than those in Mamasani, and Sepidan (*P* < 0.05 for both); however, fewer cases were seen compared with Lar (*P* < 0.05). Moreover, there were fewer cases in Lamerd and Mamasani than those in Lar (*P* < 0.05 for both). The cases were higher in Lar than those in Sepidan (*P* < 0.05).

COVID-19 cases in February, March, April, and May were significantly lower than those in June, July, August, and September (*P* < 0.05 for all, respectively); COVID-19 cases in July were significantly higher than those in August (*P* < 0.05 for all); COVID-19 cases in August were significantly lower than those in September (*P* < 0.05 for all).

### 3.2. Forecasting COVID-19 Cases in Fars Province during 1 October–14 November 2020

From the 53 554 observed cases during 18 February–30 September 2020, 55 369 cases were forecasted during 1 October–14 November 2020 using variety of time series models. The frequency of 55 369 forecasted cases alongside the results of time series analysis for the 26 cities has been presented in Table 5.

**Figure 1:**
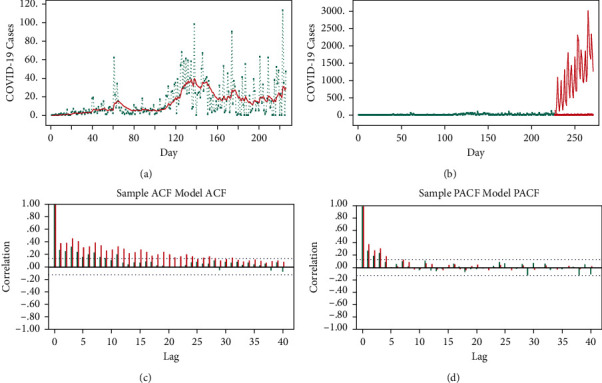
(a) SARMA_12_ (3, 4) estimating the number of COVID-19 cases in Jahrom during February^18th^–September^30th^ 2020; the green dashed line represents the number of observed COVID-19 cases during February^18th^–September^30th^ 2020; the simple red line indicates SARMA_12_ (3, 4) model estimating COVID-19 cases during February^18th^–September^30th^ 2020. (b) SARMA_12_ (3, 4) model predicting COVID-19 cases in Jahrom during October^1st^-November^14th^ 2020; the green line represents the estimated COVID-19 cases during February^18th^–September^30th^ 2020; the red line represents the predicted COVID-19 cases during October^1st^-November^14th^ 2020 with its confidence interval. (c) Sample/model SARMA_12_ (3, 4) auto correlation function (ACF) and (d) partial auto correlation function (PACF) for Jahrom during October^1st^-November^14th^ 2020; the green correlations are derived from the sample and the red correlations are taken from the fitted model; two dashed horizontal lines are confidence bands; a model is considered good in case the sample and model correlations out of the bands overlap.

**Figure 2:**
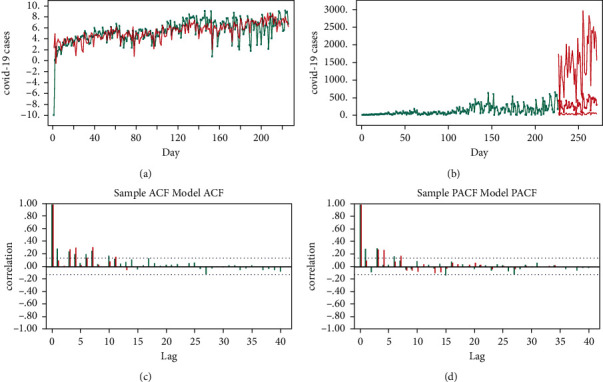
(a) MA (8) estimating the number of COVID-19 cases in Shiraz during February^18th^–September^30th^ 2020; the green dashed line represents the number of observed COVID-19 cases during February^18th^–September^30th^ 2020; the simple red line indicates MA (8) model estimating COVID-19 cases during February^18th^–September^30th^ 2020. (b) MA (8) model predicting COVID-19 cases in Shiraz during October^1st^-November^14th^ 2020; the green line represents the estimated COVID-19 cases during February^18th^–September^30th^ 2020; the red line represents the predicted COVID-19 cases during October^1st^-November^14th^ 2020 with its confidence interval. (c) Sample/model MA (8) auto correlation function (ACF) and (d) partial auto correlation function (PACF) for Shiraz during October^1st^-November^14th^ 2020; the green correlations are derived from the sample and the red correlations are taken from the fitted model; two dashed horizontal lines are confidence bands; a model is considered good in case the sample and model correlations out of the bands overlap.

From the 55 369 forecasted cases during 1 October–14 November 2020, the highest proportion was related to Shiraz (26.9%). Additionally, 65.80% and 34.20% of the cases were forecasted to occur in October and November, respectively. The time series results for Jahrom and Shiraz have been presented in Figures 1 and 2, respectively. The interpretation of the results was similar for the 24 other cities.

#### 3.2.1. COVID-19 Time Series Analysis in Jahrom during 1 October–14 November 2020

To reach the stability of variance, Box-Cox transformation was set zero. Besides, SARMA_12_ (3, 4) was fitted with the observed quadratic trend and seasonality of 12. The estimated COVID-19 cases during 18 February–30 September 2020, the predicted COVID-19 cases during 1 October–14 November 2020 (45 days), and ACF/PACF sample/model for Jahrom have been presented in Figure 1.

SARMA_12_ (3, 4) model was shown to be a good fit that can predict COVID-19 cases in Jahrom during October^1st^-November^14th^ 2020.

#### 3.2.2. COVID-19 Time Series Analysis in Shiraz during 1 October–14 November 2020

To reach the stability of variance, Box-Cox transformation was set zero. Besides, MA(8) was fitted with the observed quadratic trend. The estimated COVID-19 cases during 18 February–30 September 2020, the predicted COVID-19 cases during 1 October–14 November 2020 (45 days), and ACF/PACF sample/model for Shiraz have been presented in Figure 2.

### 3.3. Spatiotemporal Analysis

The results of the spatiotemporal analysis including 55 369 COVID-19 forecasted cases at the city level on a daily basis together with the results of subgroup analysis have been presented in Table 6.

Overall, four outbreaks occurred in Fars province during 1 October–14 November 2020, the largest and longest of which occurred in ten cities during 2–22 October, with Jahrom being the canon (MLC). Outbreaks 2 and 3 occurred in Sepidan and Fasa, respectively, and lasted from 3 to 13 November. Besides, outbreak 4 occurred in Lar and Zarrindasht on 21 October. The results of subgroup analysis demonstrated that Zarrindasht was the canon of the epidemic on 6 October.

**Figure 3:**
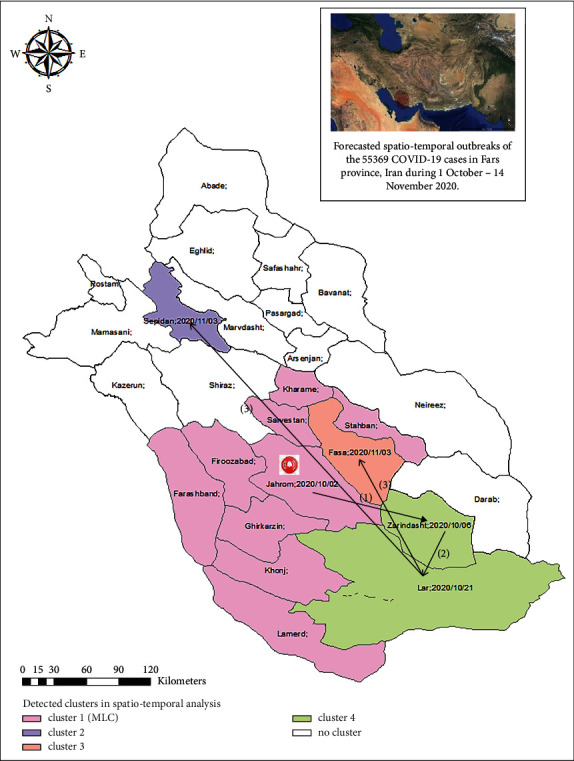
The progression of COVID-19 epidemic in Fars province, Iran during 1 October–14 November 2020.

As a part of EWS, the progression of COVID-19 epidemic resulting from spatiotemporal outbreaks of the 55 369 COVID-19 cases in Fars province, Iran, during 1 October–14 November 2020 has been shown in Figure 3.

COVID-19 epidemic inclined to trigger from Jahrom on 2 October, involving the MLC districts in the center, west, and south parts of the province; then, it showed a tendency to move towards the south in Zarrindasht on 6 October. Afterwards, it progressed to the southernmost part in Lar on 21 October. Afterwards, it simultaneously progressed to Fasa and Sepidan in the central and northwestern parts of the province, respectively on 3 November. This progression alarmed the policy makers and health managers on the potential starting point of ongoing epidemic (Jahrom city on 2 October, 2020) and, also, on when and where to prioritize the medical facilities and staffs; moreover, it could intervene with the transmission chain of COVID-19 in the area.

### 3.4. Validation Study

59 980 cases were recorded during 1 October to 14 November 2020, from which 64.2%, 5.7%, 5.5%, 2.2%, 2.2%, 2.1%, 2%, 2%, 1.8%, 1.4%, 1.2%, 1.2%, 1.1%, 1%, 1%, 0.9%, 0.8%, 0.7%, 0.7%, 0.4%, 0.4%, 0.4%, 0.4%, 0.3%, 0.3%, and 0.2% occurred in Shiraz, Jahrom, Fasa, Kazerun, Lar, Abade, Froozabad, Neireez, Sepidan, Eghlid, Stahban, Mamasani, Darab, Kharame, Pasargad, Safashahr, Bavanat, Arsenjan, Rostam, Sarvestan, Zarindasht, Farashband, Marvdasht, Ghirkazin, Lamerd, and Khonj, respectively. In addition, 51.2% and 48.8% of the cases occurred in October and September, respectively. The space-time outbreaks of the 59 980 COVID-19 incidence cases accompanied by subcluster analysis in Fars province, Iran, during 1 October to 14 November 2020 are shown in Table 7.

  Cluster 1:
  Real data: an outbreak occurred in Zarindasht (canon), Darab, and Lar during 2020/11/8–2020/11/14. Forecasted in EWS: Zarindasht and Lar (canons) were predicted to get involved in an outbreak on 2020/10/21.
  Cluster 2:
  Real data: an outbreak occurred in Fasa during 2020/11/4–2020/11/14. Forecasted in EWS: Fasa was predicted to get involved in an outbreak on 2020/11/3.
  Cluster 3:
  Real data: an outbreak occurred in Sepidan during 2020/11/13–2020/11/14. Forecasted in EWS: Sepidan was predicted to get involved in an outbreak during 2020/11/3–2020/11/13.
  Cluster 4:
  Real data: an outbreak occurred in Firoozabad (canon), Farashband, Ghirkarzin, and Sarvestan during 2020/11/8–2020/11/14. Forecasted in EWS: Firoozabad, Farashband, Ghirkarzin, and Sarvestan were predicted to get involved in an outbreak during 2020/10/2–2020/10/22.

However, Bavanat (2020/11/11–2020/11/14), Rostam (2020/11/1–2020/11/14), and Darab (2020/11/14), which were detected in validation study, were not predicted in EWS outbreaks.

The prediction of the epidemic consideration: real data showed that the epidemic was started from Jahrom on 2020/10/27 and progressed to Sarvestan, Rostam, Fasa, Zarindasht, Ghirkarzin, Farashband, Bavanat, Sepidan, Firoozabad and Darab; moreover, in EWS, the epidemic was triggered from Jahrom on 2020/10/2 and progressed to MLC districts, Zarindasht, Lar, Fasa, and Sepidan.

A total of 12 cities got involved in the outbreaks during 1 October–14 November 2020 using real data, nine of which were predicted in the EWS. The EWS predicted time periods were either the same as the observed periods or occurred earlier. Based on the validation study findings, the results of EWS were shown to be valid enough for our purpose.

## 4. Discussion

Covid-19 cases did not distributed uniformly across cities and times during 18 February–30 September 2020 in Fars province, Iran. In this protocol presented as EWS, 55 369 COVID-19 cases were forecasted from the 53 554 daily basis city-level time series data recorded during 18 February–30 September 2020. The forecasted cases resulted in the prediction of four significant spatiotemporal outbreaks of COVID-19 in the area including the MLC and three other clusters. The MLC, which was also the largest and the longest cluster, included ten cities during 2–22 October, with Jahrom being the canon. This finding, schematically presented as the progression of the epidemic during 1 October–14 November 2020, revealed that the epidemic inclined to trigger from Jahrom on 2 October, involving the MLC districts in the center, west, and south parts of the province; then, it showed a tendency to move towards the south in Zarrindasht on 6 October. Afterwards, it progressed to the southernmost part in Lar on 21 October. Later, it simultaneously progressed to Fasa and Sepidan in the central and northwestern parts of the province, respectively on 3 November. To the best of our knowledge, this protocol alarmed the surveillance system to focus on the progression of the epidemic and especially on the starting point, Jahrom, so that proper interventions could be done on the transmission chain of *SARS-CoV-2* resulting in lower prevalence of the disease in the area. Moreover, the surveillance system can dedicate more test sites, medical resources, community quarantine, vaccination coverage, and travel bans on more at risk locations at specified detected times.

Despite being the most important transmission line of the disease, it should be explained why the most populated city, Shiraz, with the maximum number of COVID-19 cases was not included in the predicted outbreaks. Shiraz was ignored in permutation scan statistics analysis, in which clusters are ranked by their likelihood ratio scores rather than by the population density or the area size. This can be attributed to the fact that, with the capital city of Fars province being with advanced medical services, many patients from nearby cities travel to Shiraz to receive health services, thereby transmitting the infection to other cities of the province.

Time series models based on statistical methods were revealed to fit well for forecasting time dependent data. They presented well-fit results in case of the COVID-19 epidemic as well. For instance, the AR model could forecast the confirmed COVID-19 cases in Iran [10]. Additionally, ARIMA-based time series analysis forecasting near future could assist the government to be prepared for the upcoming emergencies of the current epidemic in India [11–14]. In another study, ARIMA models were fitted to forecast short-term COVID-19 cases in the five most affected countries of the world including Italy, Spain, France, the United Kingdom, and the United States of America [15].

Space-time permutation scan statistics could well manage space-time dependent variables through informing the policymakers on the progression of the epidemic in China [16–18]. In the United States, the space-time outbreaks of COVID-19 were detected during two periods of time (22 January to 9 March 2020 and 18 January to 27 March 2020) using space-time statistics [19]. Space-time scan statistics also resulted in the detection of space-time outbreaks in Hong Kong during 23 January–14 April 2020 [20]. Furthermore, space-time permutation scan statistics worked better for predicting highly frequent infectious diseases with high transmission rates [8, 21, 22]. Another study employed almost the same protocol as the one used to establish the EWS in the current study in order to detect the vulnerable districts for COVID-19 in India. Using ARIMA time series model for forecasting short-term COVID-19 cases and performing spatial analysis, they indicated that the western and southern parts of the country were highly vulnerable for COVID-19 infection [23, 24].

EWS establishment could effectively warn the surveillance system about the progression of the epidemic in Fars province, Iran. However, it had several limitations, which might have affected the results. Firstly, the surveillance system of infectious diseases in Iran is a passive system, and many *SARS-CoV-2* infected people with mild to moderate symptoms did not refer to health centers and were not diagnosed. Therefore, the forecasted cases of COVID-19 might have been underestimated. This could affect estimating the required health services and medical facilities. Secondly, permutation scan statistics discover more than one cluster in a given area and time. Hence, interpretation of the results could be somewhat difficult, especially when incorporating main-cluster and subcluster results. Regarding clinical and statistical significance, prioritizing the clusters can also be difficult. Another limitation of scan statistics is that it uses a circular scan window. Thus, the precise limits of the detected clusters remain uncertain. In other words, either the site within the detected clusters may not have a high risk, or a high risk site may be out of the cluster. Yet, using subgroup analysis for detecting significant clusters within large clusters could solve the problem. Further investigations are suggested to assess the genetic variation and meteorological risk factors of COVID-19 in the long run.

## 5. Conclusion

The EWS presented here would alarm the policy makers and health managers on the potential starting point of ongoing epidemic and, also, on when and where to prioritize the medical facilities and staffs. The MLC, which was also the largest and the longest cluster, included ten cities during 2–22 October, with Jahrom being the canon. This finding revealed that the epidemic inclined to trigger from Jahrom on 2 October, involving the MLC districts in the center, west, and south parts of the province; then, it showed a tendency to move towards the south in Zarrindasht on 6 October. Afterwards, it progressed to the southernmost part in Lar on 21 October. Afterwards, it simultaneously progressed to Fasa and Sepidan in the central and northwestern parts of the province, respectively, on 3 November. To the best of our knowledge, this protocol alarmed the surveillance system to focus on the starting point and progression of the epidemic as well as to dedicate more test sites, medical resources, stricter quarantine, vaccination coverage, and travel bans on more at risk locations at specified times. These findings can be used to tailor province-level policies to servile the ongoing epidemic in the area; however, governmental level effort is needed to control the epidemic at a larger scale in the future.

## Figures and Tables

**Table 1:** Distribution of 53 554 COVID-19 cases in 26 cities of Fars province, Iran, February 18–September 30, 2020.

City	Shiraz	Lar	Jahrom	Fasa	Kazerun	Marvdasht	Darab	Abade
COVID-19 (%)	53.49	8.64	6.25	5.95	5.24	3.32	2.07	1.63
City	Zarindasht	Firoozabad	Lamerd	Mamasani	Ghirkarzin	Safashahr	Sepidan	Stahban
COVID-19 (%)	1.39	1.18	1.16	1.10	1.08	1.04	0.99	0.85
City	Eghlid	Farashband	Pasargad	Neireez	Arsenjan	Rostam	Khonj	Kharame
COVID-19 (%)	0.78	0.73	0.67	0.57	0.47	0.38	0.29	0.29
City	Bavanat	Sarvestan						
COVID-19 (%)	0.24	0.20						

**Table 2:** Distribution of 53 554 COVID-19 cases in Fars province, Iran, February 18–September 30, 2020.

Month	February	March	April	May	June	July	August	September
COVID-19 (%)	0.30	3.50	6.50	4.70	19.70	24	18.70	22.60

**Table 3:** Pairwise comparisons of 53 554 COVID-19 cases across 26 cities of Fars province, Iran, during February 18–September 30, 2020.

City	Mean difference	*P*	City	Mean difference	*P*	City	Mean difference	*P*	City	Mean difference	*P*
Shiraz-Safashahr	124.28	<0.001	Safashahr-Fasa	−11.62	<0.001	Neireez-Jahrom	−13.46	<0.001	Jahrom-Kharame	14.13	<0.001
Shiraz-Fasa	112.66	<0.001	Safashahr-Jahrom	−12.33	<0.001	Neireez-Kazerun	−11.07	<0.001	Jahrom-Farashband	13.10	<0.001
Shiraz-Neireez	125.41	<0.001	Safashahr-Kazerun	−9.95	<0.001	Neireez-Marvdasht	−6.52	0.01	Jahrom-Khonj	14.11	<0.001
Shiraz-Abade	122.9	<0.001	Safashahr-Marvdasht	−5.39	0.04	Neireez-Lar	−19.13	<0.001	Jahrom-Darab	9.91	<0.001
Shiraz-Arsenjan	125.63	<0.001	Safashahr-Lar	−18	<0.001	Abade-Jahrom	−10.95	<0.001	Jahrom-Rostam	13.90	<0.001
Shiraz-Stahban	124.73	<0.001	Fasa-Neireez	12.7	<0.001	Abade-Kazerun	−8.56	0.001	Jahrom-Zarindasht	11.1	<0.001
Shiraz-Eghlid	124.92	<0.001	Fasa-Abade	10.24	<0.001	Abade-Lar	−16.62	<0.001	Jahrom-Sarvestan	14.32	<0.001
Shiraz-Bavanat	126.18	<0.001	Fasa-Arsenjan	12.98	<0.001	Arsenjan-Jahrom	−13.69	<0.001	Jahrom-Firoozabad	12	<0.001
Shiraz-Pasargad	125.17	<0.001	Fasa-Stahban	12.08	<0.001	Arsenjan-Kazerun	−11.3	<0.001	Jahrom-Ghirkarzin	12.26	<0.001
Shiraz-Jahrom	111.95	<0.001	Fasa-Eghlid	12.25	<0.001	Arsenjan-Marvdasht	−6.7	0.01	Jahrom-Marvdasht	6.94	0.009
Shiraz-Kharame	126.08	<0.001	Fasa-Bavanat	13.52	<0.001	Arsenjan-Lar	−19.36	<0.001	Jahrom-Kharame	1.13	<0.001
Shiraz-Farashband	125.03	<0.001	Fasa-Pasargad	12.51	0.01	Stahban-Jahrom	−12.78	<0.001	Jahrom-Lamerd	12.07	<0.001
Shiraz-khonj	126.03	<0.001	Fasa-Kharame	13.42	<0.001	Stahban-Kazerun	−10.4	<0.001	Jahrom-Mamasani	12.04	<0.001
Shiraz-Darab	121.85	<0.001	Fasa-Farashband	12.37	<0.001	Stahban-Marvdasht	−5.84	0.03	Jahrom-lar	−5.67	0.03
Shiraz-Rostam	125.85	<0.001	Fasa-khonj	13.4	0.01	Stahban-Lar	−18.46	<0.001	Jahrom-Sepidan	12.46	<0.001
Shiraz-Zarindasht	123.46	<0.001	Fasa-Darab	9.19	0.001	Eghlid-Jahrom	−12.96	<0.001	Kharame-Kazerun	−11.74	<0.001
Shiraz-Sarvestan	126.27	<0.001	Fasa-Rostam	13.19	<0.001	Eghlid-Kazerun	−10.57	<0.001	Kharame-Marvdasht	−7.19	0.007
Shiraz-Firoozabad	123.96	<0.001	Fasa-Zarindasht	10.8	<0.001	Eghlid-Marvdasht	−6.02	0.02	Kharame-Lar	−19.8	<0.001
Shiraz-Ghirkarzin	124.21	<0.001	Fasa-Sarvestan	13.61	<0.001	Eghlid-Lar	−18.63	<0.001	Farashband-Kazerun	−10.69	<0.001
Shiraz-Kazerun	114.34	<0.001	Fasa-Firoozabad	11.30	<0.001	Bavanat-Jahrom	−14.23	<0.001	Farashband-Marvdasht	−6.14	0.02
Shiraz-Marvdasht	118.89	<0.001	Fasa-Ghirkarzin	11.55	<0.001	Bavanat-Kazerun	−11.8	<0.001	Farashband-Lar	−18.75	<0.001
Shiraz-Lamerd	124.02	<0.001	Fasa-Marvdasht	6.23	0.01	Bavanat-Marvdasht	−7.29	0.006	Khonj-Kazerun	−11.73	<0.001
Shiraz-Mamasani	124.15	<0.001	Fasa-Lamerd	11.36	<0.001	Bavanat-Lar	−19.9	<0.001	Khonj-Marvdasht	−7.17	0.007
Shiraz-Lar	106.28	<0.001	Fasa-Mamasani	11.50	<0.001	Pasargad-Jahrom	−13.22	<0.001	Khonj-Lar	−19.78	<0.001
Shiraz-Sepidan	124.41	<0.001	Fasa-Lar	−6.38	0.02	Pasargad-Kazerun	−10.84	<0.001	Marvdasht-Mamasani	5.26	0.04
Darab-Kazerun	−7.52	0.005	Fasa-Sepidan	11.75	<0.001	Pasargad-Marvdasht	−6.28	0.02	Marvdasht-Lar	−12.61	<0.001
Darab-Lar	−15.57	<0.001	Sarvestan-Kazerun	−11.94	<0.001	Pasargad-Lar	−19	<0.001	Marvdasht-Sepidan	5.52	0.03
Rostam-Kazerun	−11.52	<0.001	Sarvestan-Marvdasht	−7.38	0.005	Ghirkarzin-Kazerun	−9.87	<0.001	Kazerun-Lamerd	9.68	<0.001
Rostam-Marvdasht	−6.95	0.009	Sarvestan-Lar	−19.99	<0.001	Ghirkarzin-Marvdasht	−5.32	0.04	Kazerun-Mamasani	9.82	<0.001
Rostam-Lar	−19.57	<0.001	Firoozabad-Kazerun	−9.62	<0.001	Ghirkarzin-Lar	−18	<0.001	Kazerun-Lar	−8.06	0.002
Zarindasht-Kazerun	−9.13	0.001	Firoozabad-Lar	−17.68	<0.001	Lamerd-Lar	−17.74	<0.001	Kazerun-Sepidan	10.08	<0.001
Zarindasht-Lar	−17.19	<0.001	Lar-Sepidan	18.13	<0.001	Mamasani-Lar	−17.88	<0.001			

Note: std. error for all difference *s* is 2.66; just statistically significant differences were reported.

**Table 4:** Pairwise comparisons of 53 554 COVID-19 cases by month in Fars province, Iran, during 18 February–30 September 2020.

Sammonth	Mean difference	Std. error	Adj. *P*	Sample median	Mean difference	Std. error	Adj. *P*
February–June	−13.02	2.45	<0.001	April–June	−9.05	1.85	<0.001
February–July	−16	2.45	<0.001	April–July	−12.04	1.85	<0.001
February–August	−12	2.42	<0.001	April–August	−7.23	1.81	<0.001
February–September	−15.08	2.45	<0.001	April–September	−11.2	1.85	<0.001
March–June	−11.15	1.84	<0.001	May-June	−10.30	1.85	<0.001
March–July	−1.14	1.84	<0.001	May–July	−13.30	1.85	<0.001
March–August	−9.33	1.80	<0.001	May–August	−8.48	1.81	<0.001
March–September	−13.21	1.84	<0.001	May–September	−12.36	1.85	<0.001
July-August	4.81	1.81	0.008	August-September	−3.88	1.81	0.03

Note: just statistically significant differences were reported.

**Table 5:** The frequency 55 369 forecasted cases during 1 October–14 November 2020 accompanied by the results of time series analysis in the 26 cities of Fars province, Iran.

Time series analysis results												
City	Forecasted cases		Model	Residual analysis tests (*P*)						Order of min AICC YW	AIC	BIC
	*n*	%		Ljung-box	Diff sign points	Rank test statistic	McLeod-Li	Turning points	Jarque-bera			
Shiraz	14895	26.9	MA (8)	0.230	0.30	0.69	0.030	0.100	<0.001	0	8280	8303
Lar	2762	4.99	SARMA_12_ (2, 2)	0.400	0.9	0.49	0.040	0.560	<0.001	0	1098	1101
Jahrom	1716	3.10	SARMA_12_ (3, 4)	0.770	0.73	0.37	<0.001	0.010	<0.001	0	1221	1225
Fasa	1772	3.20	ARMA (1, 1)	0.460	0.73	0.12	<0.001	0.090	<0.001	0	1371	1371
Kazerun	1661	3	AR (1)	0.440	0.30	0.04	0.020	0.220	<0.001	0	1267	1266
Marvdasht	2215	4	SARMA_12_ (1, 1)	0.610	0.9	0.04	0.010	0.560	<0.001	0	1247	1247
Darab	1440	2.60	ARMA (1, 1)	0.960	0.02	0.80	0.040	0.180	<0.001	0	1360	1361
Abade	1661	3	SARMA_12_ (1, 1)	0.960	0.42	0.60	0.040	0.180	<0.001	0	1360	1361
Zarindasht	1883	3.40	AR (1)	0.860	0.03	0.85	0.010	0.950	0.030	0	1357	1356
Firoozabad	1495	2.70	SARMA_12_ (1, 2)	0.060	0.08	0.21	0.030	0.010	<0.001	0	1391	1389
Lamerd	1440	2.60	SARMA_12_ (2, 2)	0.190	0.21	0.34	<0.001	0.0002	0.030	0	1364	1368
Mamasani	1495	2.70	SARMA_12_ (1, 2)	0.580	0.04	0.32	0.040	0.490	<0.001	0	1381	1380
Ghirkarzin	1440	2.60	ARIMA (3, 1)	0.980	0.73	0.51	0.020	0.450	0.010	0	1364	1366
Safashahr	1440	2.60	ARIMA (1, 1)	0.500	0.45	0.76	0.0010	0.350	0.020	0	1348	1348
Sepidan	1993	3.60	SARMA_12_ (3, 1)	0.130	0.30	0.92	0.030	0.950	0.040	0	1344	1342
Stahban	1440	2.60	ARMA (1, 1)	0.980	0.2	0.81	<0.001	0.180	<0.001	0	1391	1392
Eghlid	1440	2.60	MA (1)	0.140	0.73	0.17	0.020	0.670	<0.001	0	1391	1388
Farashband	1440	2.60	SARMA_12_ (2, 2)	0.920	0.73	0.40	<0.001	0.040	0.001	0	1369	1370
Pasargad	1661	3	MA (1)	0.060	0.04	0.47	0.010	0.490	0.050	0	1334	1332
Neireez	1440	2.60	AR (1)	0.230	0.45	0.34	0.002	0.450	0.010	0	1364	1363
Arsenjan	1440	2.60	ARMA (2, 1)	0.080	0.42	0.14	0.030	0.830	<0.001	0	1372	1367
Rostam	1440	2.60	AR (1)	0.090	0.07	0.20	<0.001	<0.001	<0.001	0	1321	1321
Khonj	1440	2.60	SARMA_12_ (2, 1)	0.680	0.06	0.05	<0.001	0.830	<0.001	0	1295	1291
Kharame	1440	2.60	SARMA_12_ (1, 3)	0.450	0.43	0.23	0.001	0.001	0.010	0	1356	1357
Bavanat	1440	2.60	MA (1)	0.220	0.91	0.02	<0.001	0.310	<0.001	0	1308	1306
Sarvestan	1440	2.60	SARMA_12_ (2, 2)	0.540	0.9	0.96	0.010	0.590	0.130	0	1260	1258

AR, autoregressive; MA, moving average; ARMA, autoregressive moving average; ARIMA, autoregressive integrated moving average; SARMA, seasonal autoregressive moving average. It is noteworthy to mention that if four out of the six residual analysis test statistics are statistically significant, it is enough to say that the model is a good fit; based on residual analysis McLeod- Li test statistic needs to be significant and the other five tests should be greater than or equal to 0.05; in addition, the order of min AICCYW, which assesses the mean of white noise residual, needs to be zero. The lower the AIC/BIC scores, the better the model fits.

**Table 6:** The spatiotemporal outbreaks of the 55 369 COVID-19 cases in Fars province, Iran during 1 October–14 November 2020.

Cluster	Location	Radius (km)	Canon	Start date	End date	Test statistic	Critical value^†^	*P* ^*∗*^
Cluster 1 (MLC)	Ghirkarzin, Jahrom, Firoozabad, Khonj, Fasa, Farashband, Sarvestan, Lamerd, Kharame, Stahban	135.53	Jahrom	2020/10/02	2020/10/22	223.31	8.70	<0.001
Cluster 2	Sepidan	0	Sepidan	2020/11/03	2020/11/13	185.98	8.70	<0.001
Cluster 3	Fasa	0	Fasa	2020/11/03	2020/11/13	117.80	8.70	<0.001
Cluster 4	Lar, Zarindasht	76.03	Lar	2020/10/21	2020/10/21	32.28	8.70	<0.001

*Sub-cluster results*								
Cluster 4	Zarrindasht	0	Zarrindasht	2020/10/06	2020/10/09	3.23	3.08	0.04
Cluster 1 (MLC)	Fasa	0	Fasa	2020/10/06	2020/10/09	6.30	6.90	0.09
	Ghirkarzin	0	Ghirkarzin	2020/11/08	2020/11/09	3.44	6.90	0.90

^*∗*^Statistical significance was evaluated using Monte Carlo hypothesis testing at 0.05 significance level. ^†^Standard Monte Carlo critical value. MLC, most likely cluster.

**Table 7:** The space-time outbreaks of the 59 980 COVID-19 incidence cases accompanied by subcluster analysis in Fars province, Iran, during 1 October–14 November 2020.

Cluster	Location	Radius (km)	Canon	Start date	End date	Test statistic	Critical value^†^	*P* value^*∗*^
Cluster1	Zarindasht, Darab, Lar	76.03	Zarindasht	2020/11/8	2020/11/14	189.3	5.6	<0.001
Cluster2	Fasa	0	Fasa	2020/11/4	2020/11/14	138.18	5.6	<0.001
Cluster3	Sepidan	0	Sepidan	2020/11/13	2020/11/14	55.54	5.6	<0.001
Cluster4	Firoozabad, Farashband, Ghirkarzin, Sarvestan	79.70	Firoozabad	2020/11/8	2020/11/14	36.89	5.6	<0.001
Cluster5	Bavanat	0	Bavanat	2020/11/11	2020/11/14	21.47	5.6	<0.001
Cluster6	Rostam	0	Rostam	2020/11/1	2020/11/14	17.86	5.6	<0.001
Cluster7	Jahrom	0	Jahrom	2020/10/27	2020/11/14	6.72	5.6	0.02

*Sub-cluster results*								
Cluster1	Darab, Zarindasht	46.57	Darab	2020/11/14	2020/11/14	6.08	2.4	<0.001
	Zarrindasht	0	Zarindasht	2020/11/5	2020/11/14	2.1	1.53	0.01
Cluster4	Ghirkarzin	0	Ghirkarzin	2020/11/7	2020/11/14	18.23	2.71	<0.001
	Sarvestan	0	Sarvestan	2020/10/31	2020/11/14	14.58	2.71	<0.001
	Farashband	0	Farashband	2020/11/8	2020/11/14	14.25	2.71	<0.001
	Firoozabad	0	Firoozabad	2020/11/14	2020/11/14	7.61	2.71	<0.001

^*∗*^Statistical significance was evaluated using Monte Carlo hypothesis testing at 0.05 significance level. ^†^Standard Monte Carlo critical value. MLC, most likely cluster.

## Data Availability

The health service data used to support the findings of this study are restricted by the Ethics Committee of the Ministry of Health, Treatment, and Medical Education affiliated to the Shiraz University of Medical Sciences, Shiraz, Iran, in order to protect patient privacy. Data are available from Marjan Zare, Central Building of Shiraz University of Medical Sciences, Vice Chancellor for Health, fourth floor, Zand St, Shiraz, Iran, telephone number: 07132122368, E-mail: marjan.zare@gmail.com, for researchers who meet the criteria for access to confidential data.
